# Interaction between Ku80 protein and a widely used antibody to adenomatous polyposis coli

**DOI:** 10.1038/sj.bjc.6600732

**Published:** 2003-01-28

**Authors:** G T Roberts, M L Davies, J A Wakeman

**Affiliations:** School of Biological Sciences, University of Wales Bangor, Deiniol Road, Bangor, Gwynedd LL57 2UW, UK

**Keywords:** Ku80, adenomatous polyposis coli (APC), N15 antibody, crossreactivity

## Abstract

The adenomatous polyposis coli (APC) gene and its expressed product are highly studied because of its role as a tumour-suppressor protein. Inherited mutations in APC lead to the condition known as familial adenomatous polyposis (FAP), which predisposes the affected individuals to colorectal cancer. Furthermore, mutations in APC are found in the majority of sporadic cases of colon cancer. There have been many published studies concerning the cellular localisation of APC, this being fundamental to our understanding of its function, but there has also been much concern over the specificity of certain commercially available antibodies to APC. Here we report that the widely used antibody APC(N15) demonstrates a strong interaction with the Ku80 subunit of the Ku heterodimer under defined experimental conditions. Based on the data presented here, we suggest that APC(N15) is not suitable for many applications used for the study of APC.

Adenomatous polyposis coli (APC) is a large multifunctional tumour-suppressor protein. It is central to many processes including developmental regulation and it is mutated in most cases of sporadic colon cancer. Germline mutations in APC lead to the condition known as familial adenomatous polyposis (FAP), which predisposes affected individuals to colorectal cancer (Dikovskaya *et al*, 2001; Fodde *et al*, 2001; Bienz, 2002 for reviews). Much focus has recently been placed on the cellular localisation of the protein and interaction with cellular counterparts. Such studies rely on unambiguous target protein recognition by cognate antibodies.

Ku70 and Ku80 are constituent subunits of the Ku heterodimer. This protein complex, which is located primarily in the nucleus (Bertinato *et al*, 2001; Koike *et al*, 2001), has key roles in numerous cellular processes, including DNA repair, chromosome maintenance and regulation of transcription (Ramsden and Gellert, 1998). In most instances, the Ku heterodimer is in a complex with the catalytic subunit of DNA-dependent protein kinase (DNA-PKcs).

There are many commercially available antibodies that recognise epitopes of APC including APC(N15), which has been widely used for a number of applications (Henderson, 2000; Reinacher-Schick and Gumbiner, 2001; Zhang *et al*, 2001). The epitope of this rabbit polyclonal corresponds to the amino terminal 15 residues of human APC exon 1, a region identical to the corresponding mouse sequence. Manufacturers' claims suggest that this antibody is suitable for immunoblotting, immunoprecipitation (IP) and immunohistochemistry (Santa Cruz, 2000). However, the suitability of APC(N15) for immunoblotting and immunohistochemistry has been questioned (Rosin-Arbesfeld *et al*, 2001; Mogensen *et al*, 2002). We add to these concerns and present data to show that APC(N15) demonstrates a strong nonspecific interaction with the Ku80 subunit.

## Materials and Methods

### Cell lines

SW480 cells (ECACC) were grown in DMEM/10% FBS, HCT116 cells (ECACC) were grown in McCoy's 5a medium/10% FBS.

### Immunofluorescence

Cells were seeded (5×10^5^ cells) in 40 mm tissue culture dishes. Cells were washed in PBS, fixed with 4% paraformaldehyde for 20 min at RT, washed in PBS before being permeabilised in 0.2% Triton X-100 for 15 min at RT. After a further PBS wash, cells were blocked in PBS–5%FBS for 1 h at RT. Cells were incubated with primary antibodies diluted in PBS–5%FBS for 30–60 min at 37°C, rinsed in PBS (×3) for 5 min before incubation for 30–60 min at 37°C in secondary antibodies diluted in PBS–5%FBS. Following secondary antibody incubation, cells were washed (×3) in PBS and stored in PBS for imaging using a Zeiss Axioplan 2 and LSM510 confocal microscope. Antibodies and dilutions are as follows: APC(N15) 1 : 75 (Santa-Cruz Biotechnology), Ku(p80) 1 : 50 (Oncogene).

### Western blot

Whole-cell lysate was prepared (50 mM Tris-HCL, pH7.4, 100 mM Kac, 0.1% (v/v) Triton, 1 mM AEBSF, 2 *μ*g ml^−1^ aprotinin, 10 *μ*M bestatin, 10 *μ*M E64, 1 UM pepstatin, 100 *μ*M leupeptin, 1 mM sodium ortho vanadate) and cell extracts resolved by SDS-PAGE. Gels were electrophoetically transferred onto PVDF membrane. Blots were probed with antibodies diluted as follows: APC(N15) 1 : 1000 (Santa Cruz Biotechnology, California), Ku80 1 : 200 (Oncogene, Germany).

### Immunoprecipitation

Immunoprecipitation was performed essentially as described in Chan *et al* (2002). The lysis buffer used was the same as for Western blotting above. Silver staining was according to the manufacturer's instructions (Biorad, Hertfordshire, UK).

### Mass spectrometry

In-gel protein samples were automatically digested using a Micromass MassPREP Station (Micromass, Wythenshawe, UK). MS protein analysis was carried out by Micromass using electrospray MS and MS/MS on a Micromass Q-TOF2 mass spectrometer. All data were processed automatically by means of Protein Lynx software, protein identification was achieved by analysis with ProteinLynx Global Server version 1.0.

## Results

**Figure 1:**
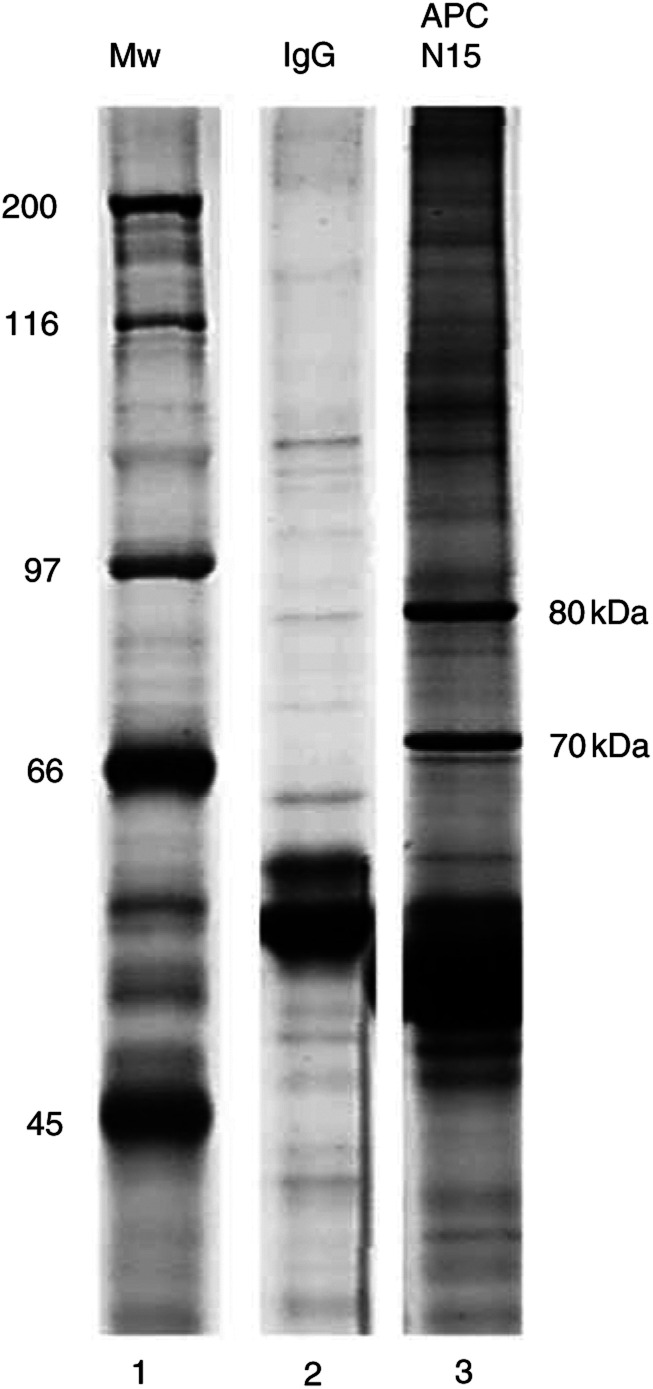
Immunoprecipitation reaction of SW480 whole-cell lysate using antibody APC(N15). Products from the IP are shown on a silver-stained SDS-PAGE gel. Lane 1 shows molecular weight markers. Two prominent bands are indicated at 70 and 80 kDa that are precipitated with the antibody APC(N15) (lane 3), but that are not present in the control lane (anti-IgG monoclonal) (lane 2).

In an effort to isolate APC and its binding partners, the antibody APC(N15) was incubated with whole-cell lysate from SW480 colon carcinoma cells. SW480 cells contain a truncated version of APC of 150 kDa as a result of a stop codon caused by frameshift mutation. Protein A–agarose beads were used to pull down the antibody–protein complex allowing protein analysis by SDS-PAGE. Following silver staining, two bands of 70 and 80 kDa showed a reproducible enrichment; however, there did not appear to be an equivalent band of the expected size for APC (Figure 1

). Both bands were excised, and analysed by mass spectrometry. The samples were unambiguously identified as an ATP-dependent DNA helicase class II 70 kDa subunit (Ku70) and an ATP-dependent DNA helicase class II 80 kDa subunit (Ku80) (Micromass, Wythenshawe, UK). Protein identifications were made by matching both peptide masses and sequences of the digested proteins (data not shown).

Adenomatous polyposis coli has been shown to interact with DNA (Deka *et al*, 1999), leading to the possibility that interaction with the Ku heterodimer may have occurred indirectly through DNA molecules with double-strand breaks. To eliminate this possibility, the IP was repeated in the presence of DNase. The removal of DNA was confirmed by running a sample on a 1% (w/v) agarose gel before visualising nucleic acid with ethidium bromide (data not shown). In the absence of DNA, Ku70 and Ku80 persistently immunoprecipitated with antibody APC(N15).

**Figure 2:**
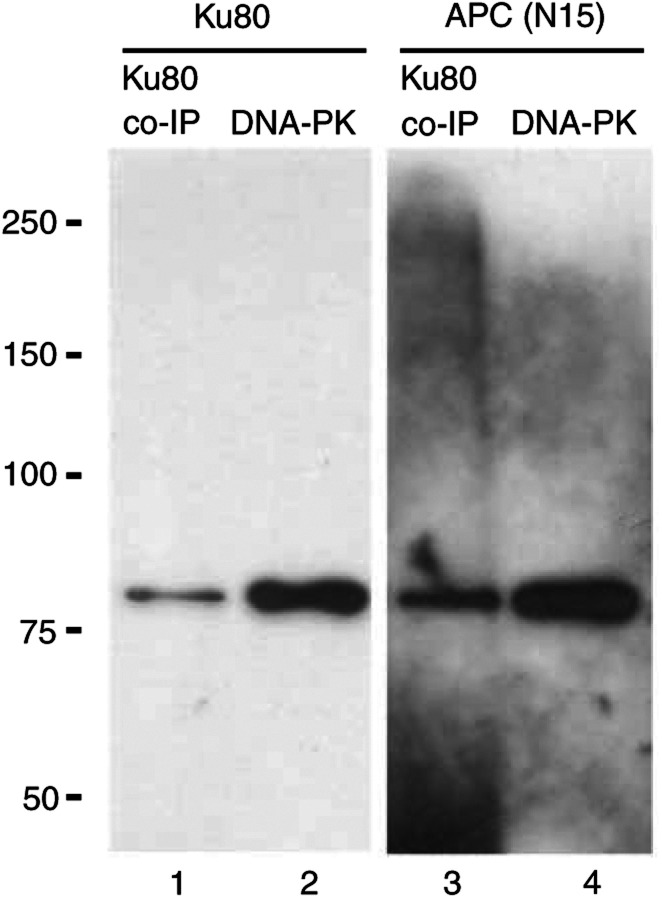
Immunoprecipitation reaction of SW480 whole-cell lysate using antibody to Ku80 followed by Western blotting using antibody to Ku80 and APC(N15). Western blots using antibodies to Ku80 and APC(N15) show bands of 80 kDa resulting from the IP with antibody to Ku80 (lanes 1 and 3). Purified DNA–PK protein complex was run alongside the products of each IP, and blotted with antibody to Ku80 and APC(N15) (lanes 2 and 4). Each of these two antibodies detects a band of 80 kDa from purified DNA–PK complex.

A final possibility to account for the coimmunoprecipitation interaction was that one or other of the Ku proteins was interacting nonspecifically with the APC(N15) antibody. To address this, an IP from SW480 whole-cell lysate was performed using an antibody to Ku80. The resulting immunoprecipitate was split into two and resolved by SDS-PAGE, each sample alongside an aliquot of purified DNA–PK complex (which includes Ku70 and Ku80), as positive control. One set was visualised by silver staining, while the other was transferred onto PVDF membrane and immunoblotted first with the Ku80 antibody and then APC(N15). The silver-stained gel revealed two enriched bands of 70 and 80 kDa (data not shown), identical in appearance to those pulled down by APC(N15). The immunoblot demonstrated that both the Ku80 and APC(N15) antibodies detected the Ku80 subunit (Figure 2

).

## Discussion

**Figure 3:**
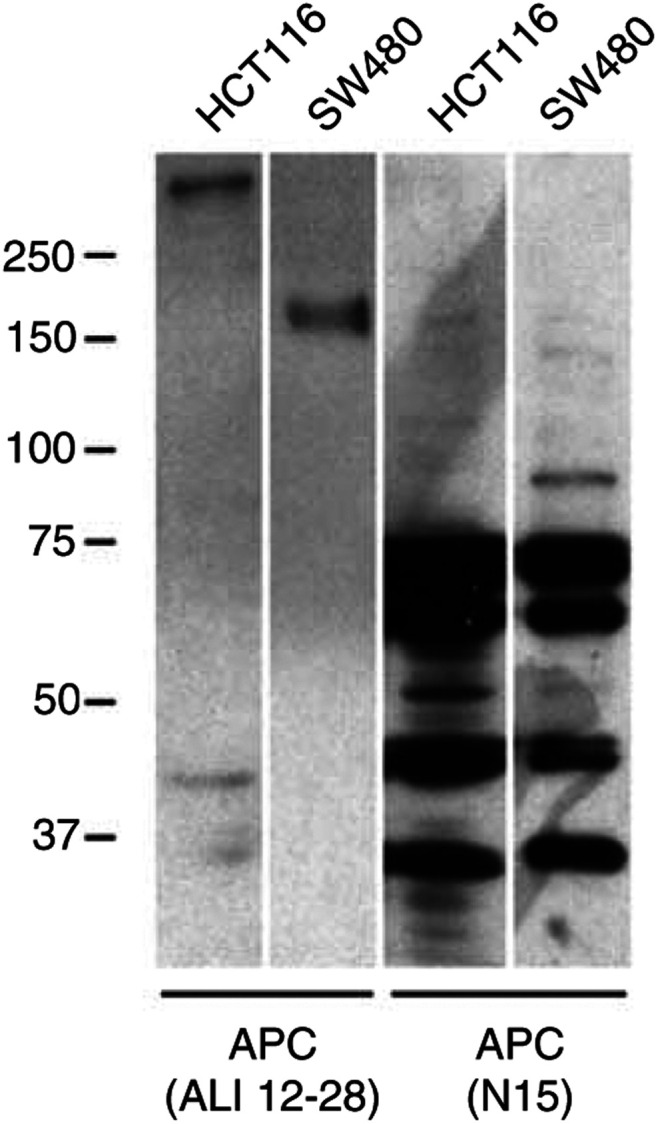
Western blot of HCT116 and SW480 whole-cell lysate using two antibodies to APC for comparison. Lane 1 shows products detected in HCT116 cells using an APC antibody (Upstate Biotechnology, Buckingham, UK). A band of the expected size for full-length APC is observed. Similarly, this antibody detects a truncated version of APC of the expected size (150 kDa) expressed in SW480 cells (lane 2). Lane 3 shows the products detected in HCT116 cells using the antibody APC(N15). Primarily, many prominent bands of smaller size are detected (the band at 80 kDa is Ku80). Although on a longer exposure a band of the predicted size for full-length APC is observed, it is also observed in SW480 cells that express truncated APC (data not shown). A faint band at 150 kDa (the expected size of this truncation) is seen in SW480 cells (lane 4), but this is also present in HCT116 cells (lane 3).

We present evidence that the widely used APC antibody, APC(N15), demonstrates a strong interaction with the Ku80 subunit component of the Ku heterodimer. This antibody is not suitable for immunoprecipitation of its intended antigen, although it is extremely efficient at affinity purifying the Ku heterodimer. As others have commented (Mogensen *et al*, 2002), a typical immunoblot with APC(N15) of whole-cell lysate reveals a number of intense bands between 40 and 90 kDa (one of these intense bands is Ku80), but only a weak band of the correct size for APC itself (Figure 3

). Furthermore, a band corresponding to the size of full-length APC was observed on longer exposures of SW480 cells, which do not contain full-length APC. This is in accordance with Mogensen *et al* (2002), who detected a similar band in DLD1 cells that also express a truncated APC protein. This indicates that the full-length band observed is most likely because of a further crossreaction.

There has been some concern as to the suitability of the widely used antibody APC(N15) for immunohistochemistry (Rosin-Arbesfeld *et al*, 2001: Mogensen *et al*, 2002), although this has yet to be fully resolved. The observation that APC(N15) does not detect ectopically expressed GFP-tagged APC or microtubule-associated APC (Rosin-Arbesfeld *et al*, 2001) adds weight to this concern.

**Figure 4:**
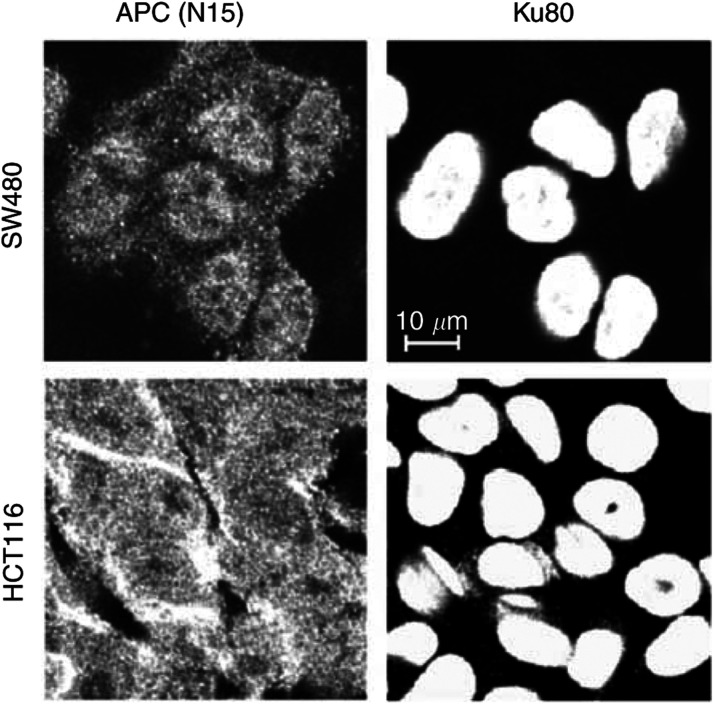
Immunofluorescence of SW480 and HCT116 cells labelled with antibody to Ku80 and APC(N15). Adenomatous polyposis coli has a predominant but not exclusive nuclear localization in SW480 cells, whereas in HCT116 cells there is strong staining in the cytoplasm as well as in the nucleus, as detected using APC(N15). Ku80 is localised exclusively in the nucleus of SW480 and HCT116 cells, as detected with Ku80 antibody.

We looked at colocalisation of APC(N15) and Ku80 (Figure 4

) and observed nuclear staining with both antibodies in SW480 and HCT116 cells. Although staining of nuclei is greater with Ku80 compared with APC(N15), it is possible that this is because of different titres of antibody. We also noted that the levels of nuclear staining with APC(N15) in SW480 and HCT116 cells are similar in contrast to the staining patterns observed using the APC antiserum raised by Nathke *et al* (1996). Finally, although APC(N15) detects additional material outside the nucleus, we cannot exclude the possibility that APC(N15) is detecting other proteins in addition to Ku80 (see immunoblot, Figure 3).

It is noteworthy that two reports published simultaneously, and describing the nuclear localisation of APC in SW480 cells (Henderson, 2000; Rosin-Arbesfeld *et al*, 2000) presented almost identical images despite one of them (Henderson, 2000) using APC(N15). One explanation could be that this presentation is fortuitous because of the abundance of nuclear APC observed in SW480 cells.

Based on the evidence we have presented here, we would suggest that APC(N15) is unreliable because of crossreactivity and is therefore not suitable for immunodetection of APC.

## Summary

The adenomatous polyposis coli (APC) gene and its expressed product are highly studied because of its role as a tumour-suppressor protein. Here we report that the widely used APC antibody (N15) demonstrates a strong interaction with the Ku80 subunit of the Ku heterodimer.
